# New design for highly durable infrared-reflective coatings

**DOI:** 10.1038/lsa.2017.175

**Published:** 2018-04-06

**Authors:** Chaoquan Hu, Jian Liu, Jianbo Wang, Zhiqing Gu, Chao Li, Qian Li, Yuankai Li, Sam Zhang, Chaobin Bi, Xiaofeng Fan, Weitao Zheng

**Affiliations:** 1 State Key Laboratory of Superhard Materials, Key Laboratory of Automobile Materials of MOE, and School of Materials Science and Engineering, Jilin University, Changchun 130012, China; 2 School of Science, Changchun University of Science and Technology, Changchun 130022, China; 3 Faculty of Materials and Energy, Southwest University, Chongqing 400715, China; 4 State Key Laboratory of Automotive Simulation and Control, Jilin University, Changchun 130025, China

**Keywords:** coating, durability, infrared reflectivity, optical design

## Abstract

The fundamental challenge in designing durable infrared-reflective coatings is achieving the ideal combination of both high reflectivity and durability. Satisfying these competing demands is traditionally achieved by deposition of durable layers on highly reflective metals. We overturn the traditional logic of ‘first reflectivity and then durability’ and propose an alternative of ‘first durability and then reflectivity’: First, a transition-metal compound is selected as a durable base; then its reflectivity is improved by incorporating silver/gold to form an alloy or by overcoating a multilayer stack. Two validation experiments prove that the new strategy works extremely well: the coatings thus obtained have infrared reflectivities close to that of aluminum, and their hardness and acid and salt corrosion resistances are 27–50, 400–1 500 and 7 500–25 000 times that of aluminum. The traditional mirror coating (e.g., Al/SiO_2_ films) is more suitable for moderate environments, while our mirror coating that was obtained by the new strategy (e.g., an Ag-doped hafnium nitride film) is more suitable for harsh environments, such as ones with dust, windblown sand, moisture, acid rain or salt fog. This work opens up new opportunities for highly durable infrared-reflective coatings and rejuvenates the study of transition metal compounds in a completely new area of optics.

## Introduction

Recently, there has been an increasing need for durable infrared-reflective coatings on large infrared telescopes^1, 2^, night-vision systems^3^, pointing and tracking systems^3^ and other optical systems^4, 5, 6, 7^. The ideal durable infrared-reflective coatings require not only high reflectivity in a particular infrared band but also durability to resist scratches and corrosion damage from long-term environmental exposure in environments with dust, windblown sand, moisture, acid rain and salt fog^8, 9^. Unfortunately, ideal durable infrared-reflective coatings are hard to obtain, as reflectivity and durability are a classical dilemma to some extent, difficult to obtain simultaneously. In Al, for example, the abundance of free electrons renders an excellent infrared reflectivity, but the electrons and the metallic bonds result in high corrosion and a low hardness, leading to a poor durability^8, 10^. In practice, Al reflective coatings are deposited in primary mirrors of a size up to 3.8 m in diameter in large infrared telescopes^1^, and those coatings have to be replaced every 2–3 years because of light scattering and reflectivity degradation resulting from scratches and corrosion. Replacing the reflectors costs extremely large amounts of time and money^9^. The sheer size of these enormous optical systems alone causes operational difficulties in the cleaning of the old coatings and the re-deposition of new coatings, thus making the process time-consuming and expensive^9, 10, 11^. Currently, achieving highly durable infrared-reflective coatings is an open and urgent challenge.

**Figure 1:**
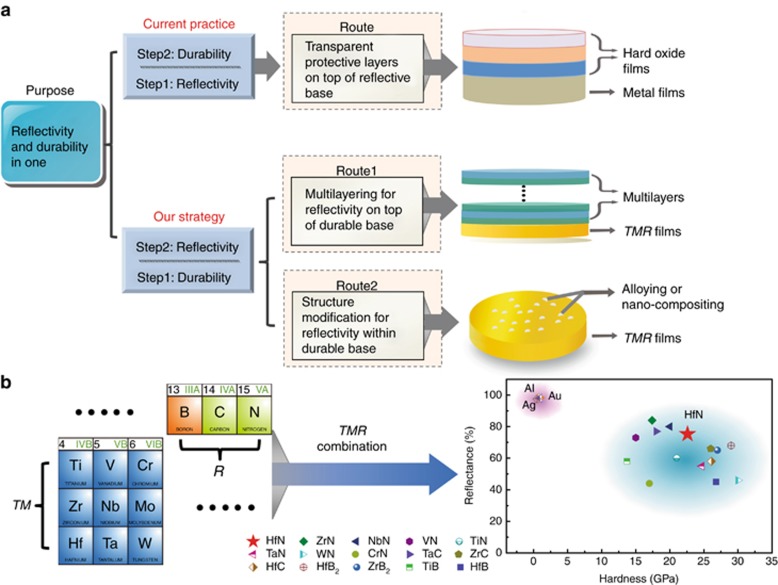
Design rules for durable infrared-reflective coatings. (**a**) The current practice and a new strategy for the combination of high reflectivity and high durability. The current practice is ‘first reflectivity and then durability’, that is, after the high infrared-reflective metal films are deposited first, transparent hard metal oxide layers are deposited on top for durability enhancement. The new strategy emphasizes ‘first durability and then reflectivity’, that is, finding a material first that satisfies the durability requirement and then modifying it to obtain a satisfactory reflectivity. The routes are to select a transition metal compound (*TMR*) as a durable base and then improve its reflectivity by overcoating a multilayer stack or by incorporating silver/gold to form an alloy or a nanocomposite, where *TMR* represents borides, carbides and nitrides of the groups IVB, VB and VIB transition metals. (**b**) Hardness^12, 13^ and infrared reflectivity^14, 15, 16, 17, 18, 19, 20, 21, 22, 23, 24, 25^ at a wavelength of 2000 nm of *TMR* as well as Al, Au and Ag, where hafnium nitride (a red pentagram) is chosen as the durable base for the validation experiments.

Traditionally, after the high infrared-reflective metal films such as Al, Ag and Au are deposited (step 1) (Figure 1a), transparent, hard metal oxide layers, such as SiO_2_ and Al_2_O_3_^26, 27^, are deposited on top (step 2) for protection, as these reflective metal films cannot withstand corrosion, scratches and other environmental damage. This route follows the typical logic of ‘achieving reflectivity first and then coating to protect’. As such, oxide films with a very high hardness^28^ are deposited on very soft metal films^12^. To avoid reflectivity loss and poor adhesion, the hard protection layer has to be very thin, usually a few dozen nanometers in thickness; thus the protection layer is often ineffective at resisting scratch damage^9, 10, 29^. Additionally, oxide films of such a thickness usually contain pinholes that undermine the protection against corrosion^10^. Therefore, the metal/oxide combination obtained from the traditional route is far from the ideal durability^8, 9, 10, 11, 29^. The traditional route also results in processing complexity because it usually involves the preparations of a variety of films to meet the multiple needs of high reflectivity, high durability and good adhesion^8, 10^.

In this paper, we overturn the traditional ‘first reflectivity and then durability’ logic and propose a new strategy of ‘first durability and then reflectivity’ (Figure 1a). Both statements of ‘first durability and then reflectivity’ and ‘first reflectivity and then durability’ refer to the design logic rather than the actual sequence during the preparation process. We recommend transition metal compounds, *TMR*s (*TM*=Ti, Zr, Hf, V, Nb, Ta, Cr, Mo, W; *R*=B, C, N), as the durable base material and then improve its reflectivity by incorporating silver/gold to form an alloy or by overcoating a multilayer stack to achieve the integration of durability and reflectivity. We use hafnium nitride (HfN_*x*_) to verify these two methods, and the experimental and theoretical results are exciting, which proves that our new strategy works wonderfully and simplistically.

## Materials and methods

### Film growth

All the films were deposited simultaneously onto optical glass and single-crystal Si (001) substrates using magnetron sputtering. The work pressure of the discharge gas was maintained at 1.0 Pa for all the deposition process. Before the deposition, the glass and Si (001) substrates were successively cleaned in acetone, alcohol and deionized water using the ultrasonic cube, and then they were introduced into the vacuum chamber (base pressure of 4 × 10^−4^ Pa) of the sputtering system. For the preparation of HfN_*x*_ films, a pure Hf target was chosen as the cathode and the mixture of N_2_ and Ar was used as the discharge gas. During the deposition, the flow rate ratios of N_2_/(Ar+N_2_) were increased from 3 to 100%, which was controlled by the mass flow controllers. The stoichiometry (*x*) of the HfN_*x*_ films was changed from 1.039 to 1.396. The other preparation parameters were maintained constant as follows. RF power applied to the Hf target: 150 W; substrate bias: −80 V; substrate temperature: 200 °C. By varying the ratios of N_2_/(Ar+N_2_), we prepared HfN-only multilayer films (see Supplementary Section 7 for the parameter selections and preparation details). Additionally, we prepared Ag-doped HfN_*x*_ films by co-sputtering the Hf and Ag target in the gas mixture of N_2_ and Ar. During the deposition, the content of Ag was controlled by changing the RF power applied to the Ag target from 0 to 100 W, while the DC power applied to the Hf target was kept constant at 150 W. Other preparation parameters were kept constant as follows. substrate bias: −160 V; substrate temperature: 200 °C; Ar flow rate: 80 sccm; N_2_ flow rate: 2.8 sccm; sample rotation rate: 5 r min^−1^. For a better comparison, an undoped HfN_*x*_ film was also prepared under the same deposition conditions, except that the RF power of the Ag target was turned off. We prepared Al/SiO_2_ films by sputtering Al and then SiO_2_ target in the discharge gas of Ar. The preparation parameters were kept constant as follows. Ar flow rate: 80 sccm; DC power applied to the Al target: 60 W; RF power to the SiO_2_ target: 100 W.

### Film characterization

A high-resolution transmission electron microscopy (JEM-2100F, JEOL, Tokyo, Japan) and grazing-incidence X-ray diffraction measurements (D8tools, Cu Kα, Bruker, Karlsruhe, Germany) were used to characterize the structures of films. An X-ray photoelectron spectroscopy (XPS, VG ESCA LAB MKII, Thermo Fisher Scientific, Waltham, MA, USA) with a monochromatized Al *Kα* (1486.6 eV) X-ray source were carried out to determine the stoichiometry *x*, core-level spectra and valence band spectra of the HfN_*x*_ films. Before the measurement of XPS, all the samples were subjected to a 180 s Ar^+^ cleaning procedure to remove the surface carbon and oxygen. A Dektak surface profiler and four-point probe measurements were used to determine the thickness *d* and DC resistivity *ρ* of the films, respectively. A UV-visible spectrometer (Lambda 950, Perkin Elmer, US) was employed to obtain UV-visible reflectivity and transmission spectra, and an FTIR spectrometer (Perkin Elmer Spectrum One B type, Perkin Elmer, US) was employed to measure infrared reflectivity spectra. By analyzing the transmission spectra, we obtained the refractive indices and absorption coefficients, using a procedure reported by Swanepoel^30^. According to the Tauc equation, we plotted (*αhν*)^2^ against the photon energy *hν* and calculated the optical gaps. An nanoindenter (MTS XP, MTS, US) was used to evaluate the hardness, where a continuous-stiffness-measurement mode was used. Hall-effect measurements (HL5550) were performed to obtain the concentrations of the free electrons of the films. An energy dispersive spectrometer equipped in a field-emission scanning electron microscope (SU8010, Hitachi, Tokyo, Japan) was used to determine the chemical composition of the Ag-doped HfN_*x*_ films. The corrosion behaviors of the samples were measured using the Tafel curves in acid and base media corresponding to 0.5 mol l^−1^ H_2_SO_4_ and 3.5 wt.% NaCl solutions, respectively. An electrochemical workstation (CHI660E) was used to perform the corrosion measurements, which was connected to a three-electrode electrochemical reactor. The working electrode, reference electrode and auxiliary electrode were the coated samples, calomel and a Pt sheet, respectively. Before the measurements, the samples were tested in an open circuit potential mode for 6 min with a scanning rate of 1 mV s^−1^. Furthermore, the corrosion behavior of the films in a seawater environment was evaluated using salt-bath experiments. The samples were immersed in a 5.0 wt.% NaCl solution and incubated at 35±1 °C for 1, 5, 10, 15, 30, 60, 120 and 180 min and 10 days (14 400 min). A reflection fluorescence microscopy (DM 2500M, Leica, Wetzlar, Germany) was employed to observe surface corrosion morphology.

### First-principles calculations

The present calculations were performed by the method of projector augmented-wave pseudopotentials with density functional theory coded in the Vienna ab inito simulation package^31, 32^. For the electrons’ exchange correlation energy, the Perdew–Burke–Ernzerhof function was used^33^. The kinetic energy cutoff is chosen as 550 eV for the plane wave expansion. The Brillouin zones were sampled with Monkhorst–Pack method. In order to make sure the convergence of total energy at 1 meV per atom level, the Γ-centered high-density k-point grid sets were chosen. In the calculation, we have considered the effect of spin polarization. Detailed modeling process can be seen in Supplementary Sections 3,4 and 10.

## Results and discussion

### Optical design for achieving highly durable infrared-reflective coatings

To achieve highly durable infrared-reflective coatings, we propose a new strategy of ‘first durability and then reflectivity’ (Figure 1a), in other words, first finding a material that satisfies the durability requirement and then modifying it to obtain the required infrared reflectivity. These two steps are illustrated in detail as follows.

#### Step I. Selection of a durable base

A high concentration of free electrons in a material induces a large plasma energy (Equation (1)), which in turn affects the dielectric function (Equation (2)). The relationship between the reflectivity and dielectric function can be described by (Equations 2, 3, 4, 5) Refs. 34, 35, 36, 37,


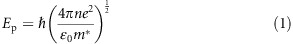



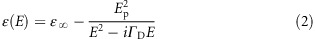



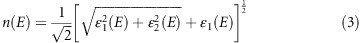



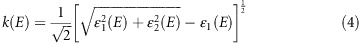



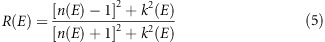


where the functions *ε*(*E*), *n*(*E*), *k*(*E*) and *R*(*E*) represent the complex dielectric function, refractive index, extinction coefficient and reflectance versus photon energy *E*, respectively; the variables *n*, m*, *ε*_0_, *ε*_∞_, *E*_p_ and Γ_D_ represent the concentration of free electrons, effective electron mass, vacuum permittivity, background constant, plasma energy and relaxation energy, respectively; and *ε*_1_(*E*) and *ε*_2_(*E*) are the real part and the imaginary part of *ε*(*E*), respectively. According to these Equations, 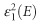 and 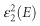 increase with *E*_p_ (Equation (2)), which induces an increase in *n*(*E*) and *k*(*E*) (Equations (3 and 4)) and a subsequent increase in *R*(*E*) (Equation (5)). The proportional relationship above between *E*_p_ and *R*(*E*) has been well demonstrated by previous simulations and experiments^38^. Hence, a sufficiently high concentration of free electrons is necessary for a high infrared reflectivity; metals such as Al, Ag and Au are good examples^37, 39^. A durable material is usually associated with high hardness, which is, in essence, proportional to the degree of covalent bonding and the bond strength^40^. Therefore, to achieve a high durability and a high reflectivity, the ideal candidate should contain strong covalent bonds and a high concentration of free electrons. The borides, carbides and nitrides of groups *IVB* (*d*^2^*s*^2^), *VB*(*d*^3^*s*^2^) and *VIB* (*d*^4^*s*^2^) transition metals, hereinafter referred to as *TMR*s, where *TM*=Ti, Zr, Hf, V, Nb, Ta, Cr, Mo and W and *R*=B, C and N, have not only strong *TM*–*R* quasi-covalent bonds from the hybridization between the *R*_p and *TM*_d orbitals but also high concentrations of unbound *d*-orbital free electrons. The strong quasi-covalent bonds endow *TMR*s with a superior durability, including high bulk moduli^41^, high hardness^42, 43, 44, 45, 46^, high melting point^47, 48, 49^ and corrosion and abrasion resistance^50, 51, 52, 53^, and makes them well known as cutting-tool coating materials^54, 55, 56^. The presence of unbound *d*-orbital free electrons causes *TMR*s to have similar electrical properties and infrared reflectivity characteristics to pure metals^57^, causing them to be widely used in the fields of superconducting materials^58, 59^ and optoelectronics^60, 61^. From these studies, it is known that *TMR*s have high durability and metal-like reflective characteristics (Figure 1b); thus *TMR*s are a category of ideal candidates to achieve the ultimate aim of both a high reflectivity and a high durability.

#### Step II. Reflectivity enhancement

From Figure 1b, the hardness of a *TMR* (14–30 GPa) is much higher than that of the reflective metals Al, Ag and Au (~0.5 GPa), but their reflectivity (40–80%) is far below that of these metals (90–98%). This explains why *TMR*s were basically excluded from the ‘radar screen’ in the search for infrared-reflective coatings. How to significantly increase the reflectivity of a *TMR* will be the key to solving the issue of needing a high durability and a high reflectivity. Taking advantage of the structural characteristics of *TMR*s, we propose two methods to improve the reflectivity of *TMR* films. One method is to deposit multilayer films on a durable *TMR* base to boost the reflectivity (Figure 1). These multilayer films are obtained by alternately depositing a transparent layer *A* and a transparent layer *B* on a durable *TMR* film, namely, *TMR*/*A*/*B*/... */A*/*B*/ (determination of the number of layers is discussed later). According to the optical interference principle^62, 63^, the multilayers can achieve a very high reflectivity close to 100% in the vicinity of a target wavelength when the refractive index of layer *A* is far less than that of layer *B*, and the optical thickness *nd* (where *n* is the refractive index and *d* is the film thickness) of layer *A* and layer *B* both equal a quarter of the target wavelength. The other method is to introduce a metal with a high concentration of free electrons (e.g., Ag, Au) into a *TMR* film to form a metal–*TMR* alloy or a metal–*TMR* nanocomposite (Figure 1a). With a high concentration of free electrons, the alloys and nanocomposites are expected to have high reflectivities over the whole infrared range. Based on this principle, we incorporate gold or silver into a *TMR* film and explore the relationship among the composition, structure, reflectivity and durability.

### Verification experiments for achieving highly durable infrared-reflective coatings

From Figure 1b, the near-stoichiometric HfN films (HfN_*x*_, *x*=N:Hf) in a rock salt structure have not only a high infrared reflectance of 75% but also a high hardness of 22.6 GPa, very close to idealistic highly durable infrared-reflective coatings. Near-stoichiometric HfN_*x*_ is thus our first choice as the base material to carry out the below mentioned two aspects of studies to boost infrared reflectivity: (I) depositing multilayers on top of the HfN_*x*_ film to boost the infrared reflectivity at a single wavelength; (II) doping silver into the HfN_*x*_ film to boost the infrared reflectivity over a wide range of wavelengths.

#### Achieving highly durable infrared-reflective coatings for a specific wavelength through multilayering

**Figure 2:**
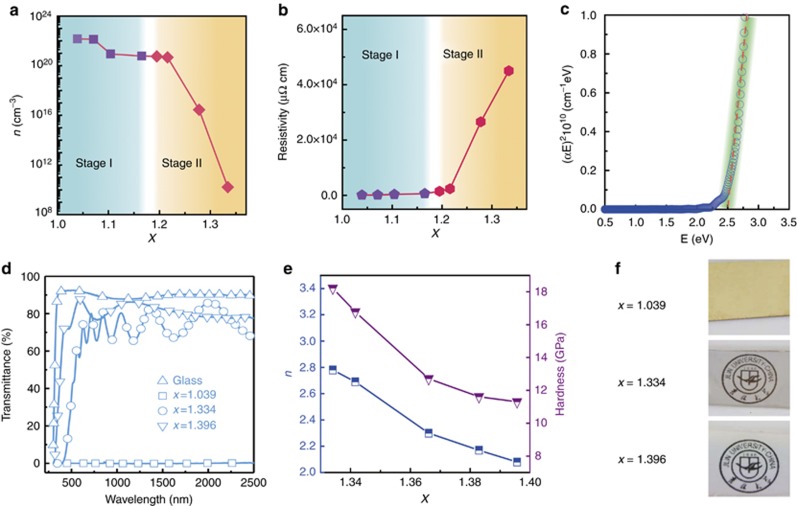
Electron localization in hafnium nitride films and its influence on the optical properties. (**a**–**c**) Electron concentration **a**, resistivity **b** and optical gap **c** of HfN_*x*_ films with different stoichiometries *x*, by which the two-stage electron localization from slow to fast is shown. (**d**–**f**) The transmission spectra **d**, the refractive index and hardness **e** and appearance **f** of HfN_*x*_ films with different stoichiometries *x*, where the three optical characteristics are revealed: an opaque metal (*OM*, *x*=1.039), a high-refractive-index transparent semiconductor (*HT*, *x*=1.334–1.342, *n*=2.78–2.69) and a low-refractive-index transparent semiconductor (*LT*, *x*=1.383–1.396, *n*=2.17–2.08).

To verify the reflectivity boost at a specific target wavelength, we develop a novel homogeneous multilayer film with three significant optical characteristics consisting entirely of HfN_*x*_. The creation of this multilayer originates from the unique behavior of electron localization in HfN_*x*_ and the resulting tunable reflectivity/transmission properties. According to the measurements of the electron concentration (Figure 2a), the resistivity (Figure 2b) and the optical gap (Figure 2c), we find that, as the N/Hf ratio increases from 1:1 (a measured *x* value of 1.039) to 4:3 (a measured *x* value of 1.334), the free electrons of HfN_*x*_ films are completely localized and the films transform from a metal to a semiconductor with an optical gap of approximately 2.5 eV (see Supplementary Section 1 for more detailed discussion). Our electron concentration (Figure 2a) and resistivity measurements (Figure 2b) show that the electron localization goes through two stages of transition from slow to fast. In the first stage, the electron concentration decreases slowly from 1.46 × 10^22^ to 6.01 × 10^20^ cm^−3^ (or a decrease of <2 orders of magnitude) as *x* increases from 1.039 to 1.165 (over a range of 0.126 in *x*), and the electrical resistivity gradually increases from 110 to 636 μΩ cm (or <6 times). In the second stage, as *x* further increases from 1.195 to 1.334 (over a range of 0.139 in *x*), the electron concentration decreases sharply from 5.59 × 10^20^ to 1.67 × 10^10^ cm^−3^, a decrease of 10 orders of magnitude. The electrical resistivity sharply increases 30 times from 1.51 × 10^3^ to 4.50 × 10^4^ μΩcm. These results demonstrate that the electron localization in HfN_*x*_ films experiences two stages as *x* increases: *x*=1.039–1.165, electrons are ‘gradually’ localized; *x*=1.195–1.334, a small increase in *x* causes a large number of electrons to be localized, resulting in the films losing their metallic characteristic and ‘rapidly’ transform into semiconductors. The microscopic origin of the two stages is completely different. In the first stage, the formation of Hf vacancies enables partial localization of free electrons around the Fermi level and promotes new localized states from N_p, which is confirmed by a good agreement among the calculated density of states (DOS) near the Fermi level (Supplementary Fig. S3a), the distribution of electron density differences (Supplementary Fig. S3b) and the measured XPS core-level spectra (Supplementary Fig. S3c). In the second stage, a phase transition from rock salt HfN to cubic Hf_3_N_4_ occurs and causes the complete localization of free electrons and the creation of new hybridized states from Hf_d and N_p. This is proven by the calculated DOS (Supplementary Figure S3d), the distribution of the electron density difference (Supplementary Fig. S3e) combined with the measured XPS valence band (Supplementary Fig. S3f) and the core-level spectra (Supplementary Fig. S3c). The Hf vacancy and phase transition in the film are identified via high-resolution transmission electron microscopy, selected area electron diffraction, Raman, X-ray diffraction and XPS (see Supplementary Section 2). All the results support each other, proving that the structures are different in the two stages. In the region of *x*=1.039–1.165, the increase in *x* is compensated by the increasing formation of Hf vacancies, while the rock salt structure remains. In the region of *x*=1.195–1.334, the further increase in *x* cannot be balanced by the Hf vacancies, and formation of the *c*-Hf_3_N_4_ phase occurs. When N/Hf=4:3 or *x* reaches 1.334, this phase transition is complete. Detailed discussions of the mechanisms of electron localization are given in Supplementary Sections 2-4.

Understanding the electron localization process, we explore the effect of electron localization on the optical properties of the films. We determine from the transmission spectra (Figure 2d) that before the electron localization, the HfN_*x*_ film (*x*=1.039) is completely opaque, a typical metal-like characteristic, so that the transmittance of this sample is zero and there are no interference fringes. After the electron localization (*x*=1.334 and 1.396), the films transform into transparent semiconductors, and thus interference fringes occur in the range of 500–2 500 nm (Figure 2d). According to the Equation 2*nd*=*mλ* (^ref.^30^^), a larger optical thickness *nd* (where *n* is the refractive index and *d* is the film thickness) produces more interference fringes. Therefore, the interference fringes of the film with *x*=1.334 (*nd*=2.78 × 625 nm) are more pronounced than those of a film with *x*=1.396 (*nd*=2.08 × 217 nm) due to the film’s larger refractive index and film thickness. Furthermore, the refractive index is found to decrease from 2.78 to 2.08, while the extinction coefficient remains approximately 0 (<0.001) as *x* increases further from 1.334 to 1.396 (Figure 2e), which is attributed to the decrease in the average molecular polarizability of the film because the polarizability of N atoms (*α*_N_=1.1 × 10^−24^ cm^3^) (^ref.^64^^) is much smaller than that of Hf atoms (*α*_Hf_=15.3 × 10^−24^ cm^3^) (^ref.^65^^). These results suggest that the optical characteristics of the HfN_*x*_ films are easily controllable by changing the stoichiometry *x* (Figure 2f): if *x* is approximately 1, the film is an opaque metal (*OM*); if *x*=1.334–1.342, the film is a high-refractive-index transparent semiconductor (*HT*, *n*=2.78–2.69); if *x*=1.383–1.396 the film is a low-refractive-index transparent semiconductor (*LT*, *n*=2.17–2.08).

**Figure 3:**
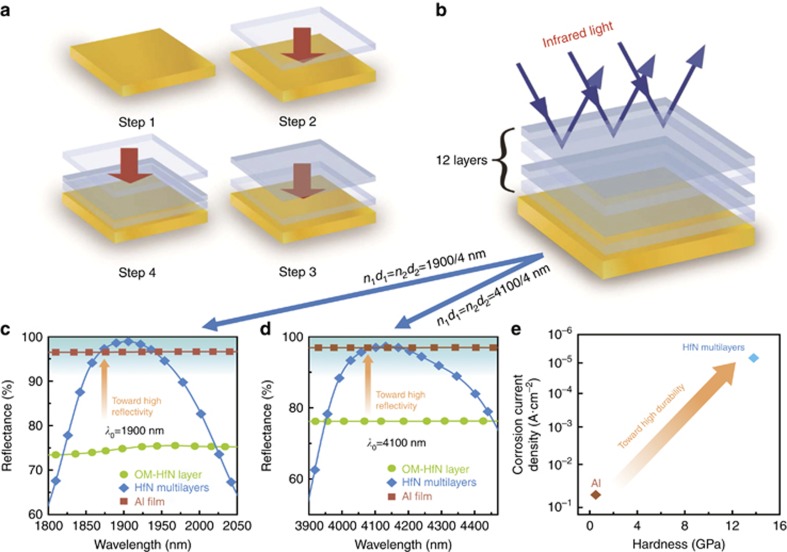
Design and one-step preparation of multilayer films of HfN_*x*_. (**a** and **b**) Design of multilayers consisting of hafnium-nitride-only films: *OM* (Step 1)/*LT* (Step 2)/*HT* (Step 3)/*LT* (Step 4)/*HT*/*LT*/*HT*. (**c** and **d**) The infrared reflectivity spectra for the *OM* single layer, multilayer and Al films. By changing the optical thickness *nd* (where *n* is the refractive index and *d* is the film thickness) to be 1900/4 nm and 4100/4 nm, a higher infrared reflectivity than that of Al is achieved in the targeted wavelengths of 1900 and 4100 nm, respectively. For the multilayers with *λ*_0_=1900 nm, the refractive indices and the thicknesses of the LT and HT layers are *n*_1_=2.08, *d*_1_=228 nm and *n*_2_=2.69, *d*_2_=177 nm, respectively. For the multilayers with *λ*_0_=4 100 nm, *n*_1_=2.17, *d*_*1*_=472 nm and *n*_2_=2.78, *d*_2_=369 nm. (**e**) The hardness and corrosion current density in a 3.5 wt.% NaCl solution for the multilayer and Al films, which indicate the multilayer film possesses a much higher durability than the Al film.

Using this tunability of the reflectivity/transmission properties, we develop multilayers consisting of hafnium-nitride-only films: *OM*/*(LT*/*HT)*_*z*_, where *z* is the total number of repeating layers (Figure 3a and 3b). For the sake of simplicity, we refer to this structure as a ‘multilayered film’. The reflectivity of a multilayered film depends on the total number of repeating layers. According to the principle of optical interference, the more repeating layers there are (i.e., the higher the value of *z*), the better the reflectivity enhancement is. However, increasing the number of repeating layers increases the deposition time and the processing difficulty. Everything considered, we set the total number of repeating layers as high as 6. Thus a periodic *LT*/*HT* stack with *z*=6 is designed (see Supplementary Section 5). For the multilayers with *λ*_0_=1 900 nm, the refractive indices of the LT and HT layers are *n*_1_=2.08 at *x*=1.396 and *n*_2_=2.69 at *x*=1.342, respectively. According to *n*_1_*d*_1_=*n*_2_*d*_2_=*λ*_0_/4, when *λ*_0_=1900 nm, the thickness of the two layers are *d*_1_=228 nm and *d*_2_=177 nm. Similarly, for *λ*_0_=4100 nm, *n*_1_=2.17 at *x*=1.383 and *n*_2_=2.78 at *x*=1.334, *d*_1_ and *d*_*2*_ are 472 and 369 nm, respectively (see Supplementary Section 6 for more details). To verify the above designs, we prepare the multilayered film of *OM*/*(LT*/*HT)*_6_ and find that the reflectivity of the *OM* layer improves tremendously (Figure 3c and 3d). We obtain experimentally the refractive index of each layer by controlling the composition of the films according to the relationship between the refractive index and the composition (Figure 2e). We obtain the desired thickness of each film by adjusting the deposition time. The detailed deposition parameters for the two multilayered films are given in Supplementary Tables S1 and S2 in Supplementary Information (See Supplementary Section 7). By controlling the optical thickness *nd* of the *LT* and *HT* layers, we achieve an infrared reflectivity higher than that of Al at a targeted wavelength *λ*_0_. For example, when both *n*_1_*d*_1_ and *n*_2_*d*_2_ are equal to 1900/4 nm, the reflectivity of the multilayers is 99.0% at 1900 nm, exceeding that of the pure Al film (96.5% at 1900 nm) (Figure 3c). When both *n*_1_*d*_1_ and *n*_2_*d*_2_ are equal to 4100/4 nm, the reflectivity is 97.0% at 4100 nm, again higher than that of the pure Al film (96.8% at 4100 nm) (Figure 3d).

**Figure 4:**
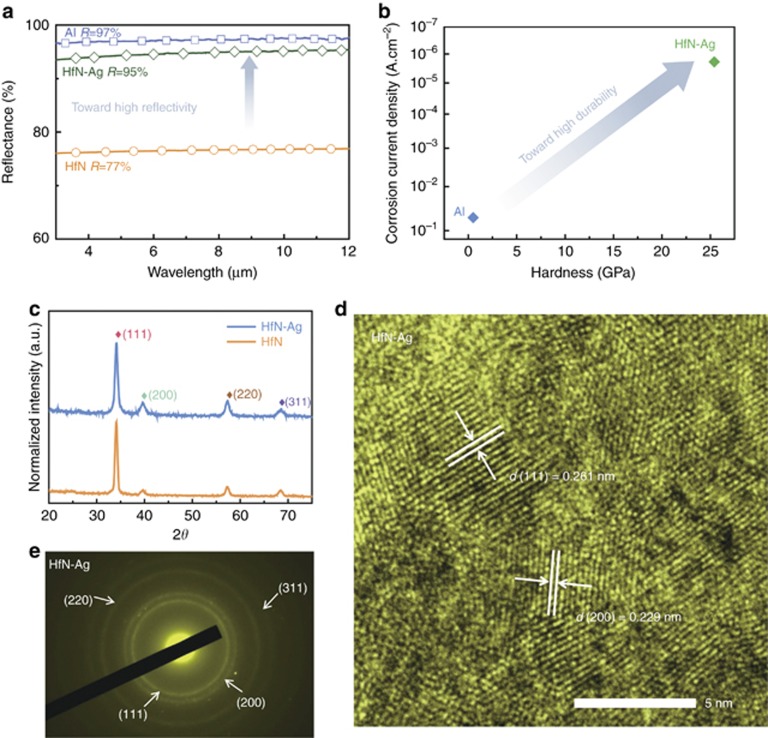
Properties and structure of Ag-doped HfN films. (**a** and **b**) The infrared reflectivity spectra **a** and hardness and corrosion current density in a 3.5 wt.% NaCl solution **b** for the Ag-doped HfN (930 nm), HfN (886 nm) and Al films (858 nm), where the Ag-doped HfN film has a high durability and a high infrared reflectivity. (**c**–**e**) The grazing incident X-ray diffraction pattern for the Ag-doped HfN film and HfN films **c**, the high-resolution transmission electron microscopy **d** and selected area electron diffraction **e** for the Ag-doped HfN film. These results prove the formation of an Ag-doped HfN film solid solution.

As an indication of durability, we characterize the hardness and the corrosion behavior of the multilayered film (see Supplementary Section 8). Because Al is the most commonly used infrared-reflective coating material^8, 9^, we conduct the same tests on an Al film as a comparison. The hardness of the multilayered film is 13.8 GPa, 27 times beyond that of the Al film (0.5 GPa) (Figure 3e). In a 0.5 mol l^−1^ H_2_SO_4_ solution, the corrosion current density of the multilayered film is 2.94 × 10^−6^ A cm^−2^, three orders of magnitude less than that of the Al film (4.69 × 10^−3^ A cm^−2^) (Supplementary Figure S4). In other words, the acid corrosion resistance of the multilayered film is >1500 times better than that of Al. In a salt solution (3.5 wt.% NaCl), the corrosion current density of the multilayered film is 6.76 × 10^−6^ A cm^−2^, in contrast to that of the Al film of 5.26 × 10^−2^ A cm^−2^ (Figure 3e and Supplementary Figure S5). This fact means the multilayered film is 7500 times more corrosion resistant than Al in this salt solution. Additionally, during salt-bath experiments (see Supplementary Section 9), the Al film shows significant corrosion after immersion in a NaCl solution at 35 °C for 5 min. When the duration time gradually increases to 180 min, the corrosion pits continue to increase (Supplementary Fig. S6). However, the multilayered film does not show any corrosion traces in the whole 10 days (14 400 min) of the salt-bath experiment (Supplementary Fig. S6), indicating that the corrosion resistance of the multilayered film in a seawater environment is easily 3000 times that of Al. These results demonstrate that the HfN_*x*_-based multilayer film possesses a much higher scratch and corrosion resistances than pure Al. In conclusion, by exploiting the tunable properties between reflectivity and transmission induced by electron localization, we successfully achieve HfN_*x*_-only multilayer films with both high infrared reflectivity and high durability. Compared with the conventional multilayer stacking of a variety of materials, our approach involves only one material (HfN_*x*_) with switching of the optical states achieved by changing the stoichiometry *x* (i.e., via the partial pressure of nitrogen only), thus greatly simplifying the manufacturing process. Additionally, as seen in Figures 3d and 4a, the reflectance (97%) of the multilayered HfN film at 4100 nm is higher than that of the Ag-doped HfN film (94%) at the same wavelength. This means that a multilayered HfN film has better reflective properties than an Ag-doped HfN film at a target wavelength or in a narrow band, which is very useful for many important optical applications (e.g., pointing and tracking optical systems^3^).

#### Achieving highly durable infrared-reflective coatings over a range of wavelengths through silver doping

To verify the reflectivity boost over a range of wavelengths, we dope Ag into near-stoichiometric HfN_*x*_ film using co-sputtering. For simplicity, we refer to the Ag-doped HfN film as ‘the doped film’. We investigate the effect of the Ag content (*C*_Ag_) on the structure and reflectivity. Our reflectivity measurements show that the average reflectance of the Ag-doped films in the range of 3–12 μm increases from 77 to 95% as the Ag content increases from 0 to 3.1%. However, when the Ag content further increases to 3.9%, the reflectance reduces to 80% due to an increase in the surface roughness. Additionally, it is found that the hardness of the films increases from 22.6 GPa to 25.4 GPa to 32.4 GPa as the Ag content increases from 0% to 3.1% to 3.9%. However, the hardness decreases significantly when more Ag is incorporated. Considering that the film with the Ag content of 3.1% has the highest infrared reflectivity and a relatively high hardness, we believe that the Ag doping of 3.1% is the best. Figure 4a shows the infrared reflectivity spectra of pure HfN (*C*_Hf_=50.6%, *C*_N_=49.4%) and the doped film (*C*_Ag_=3.1%, *C*_Hf_=50.2%, *C*_N_=46.7%) together with that of an Al film as reference. The reflectivity of a pure HfN film is only 77%, while that of the doped film increases abruptly to 95%, close to that of an Al film (97%) over a spectrum of 3 to 12 μm.

**Figure 5:**
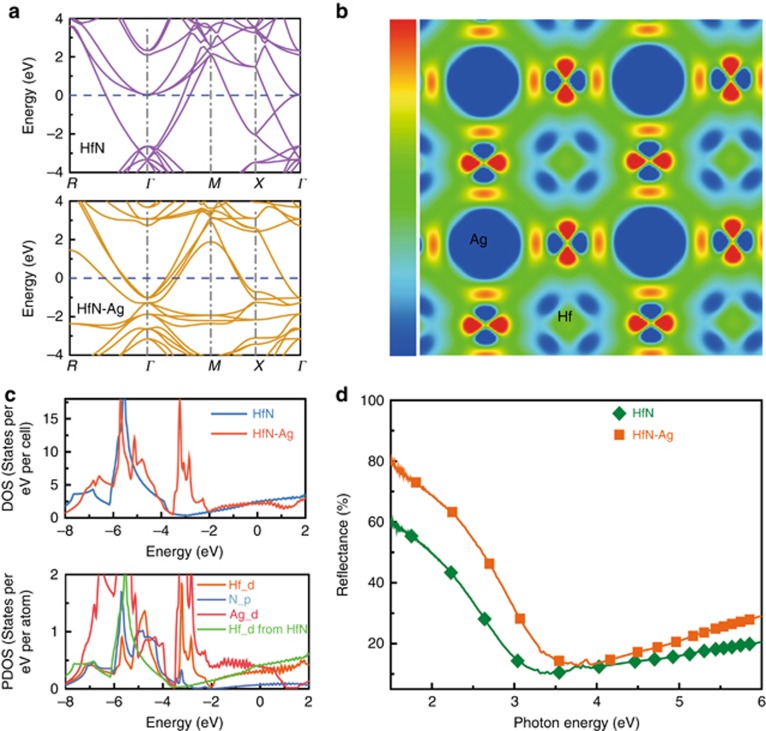
Electronic structure of Ag-doped HfN films. (**a**) Band structures of Ag-doped HfN film and HfN obtained by first-principles calculations. (**b**) 2-D distribution of the electron density differences for an Ag-doped HfN film calculated using the formula Δ*Chg (x)*=*Chg*_Ag-doped HfN film_ *(x)*−*Chg*_HfN:VHf_ *(x)*−*Chg*_Ag_ *(x)*, where *Chg*_Ag-doped HfN film_ *(x)*, *Chg*_HfN:VHf_ *(x)* and *Chg*_Ag_ *(x)* are the real space distributions of the charge densities of an Ag-doped HfN film, HfN with the Hf vacancy (V_Hf_) and an Ag atom. (**c**) DOS and partial DOS (PDOS) of an Ag-doped HfN film and HfN. (**d**) The UV-visible reflectivity spectra for the Ag-doped HfN film and HfN films, which indicate that the plasma energy of the two samples are 3.0 and 2.6 eV, respectively. These results show that the enhancement of the reflectivity is attributed to the increase of the plasma energy.

To explore the cause of the reflectivity enhancement, we characterize the structure of the samples. Figure 4c shows the grazing incident X-ray diffraction pattern for the pure and doped films, wherein both samples contain diffraction patterns attributed to the rock salt phase, showing that the pure and doped films have the same rock salt structure. Figure 4d shows lattice images from high-resolution transmission electron microscopy of the doped film, in which well-crystallized nanograins are uniformly distributed on the film surface. The measured interplanar spacings agree well with the (111) and (200) plane spacings of the rock salt phase. These findings are consistent with the selected area electron diffraction (Figure 4e), indicating the formation of a HfN(Ag) solid solution upon the doping of the Ag. A model of the HfN(Ag) solid solution with the replacement of Hf atoms by Ag atoms is constructed (see Supplementary Section 10), and first-principles calculations are performed to investigate the effect of the Ag introduction on the electronic structure. The results from the band structure of the HfN and Ag-doped HfN films (Figure 5a) show that the doped Ag does not introduce defect states near the Fermi level. From the DOS (Figure 5c), the concentration of the conductive electrons (from the contribution of the DOS near the Fermi level) does not have an obvious change. This is consistent with the measured resistivity of the pure (5.4 Ω) and doped films (5.3 Ω). Additional electron states among −3.5 eV and −2 eV are introduced by the doping (Figure 5c). These extra electrons induce the charge polarization near N atoms (Figure 5b). From the band structure (Figure 5a), these extra states have a good dispersion due to the hybridization with the nearby N atoms. They have an important contribution from the free electrons to the energy of the plasma. The visible-near-infrared reflectivity spectra (Figure 5d) shows that the plasma energy of a HfN film increases significantly from 2.6 to 3.0 eV after the introduction of Ag, which agrees well with the calculation above. Previous studies^66^ have shown that the reflectivity of HfN films in the visible-infrared band depends on both the intraband transition that is related to the free electrons (described by a Drude part) and the interband transition that is related to the bound electrons (described by a Lorentz part). The reflectivity in the infrared region depends mainly on the intraband transition and is closely related to the plasma energy. The Ag-doping-induced increase in the infrared reflectivity is attributed to an increase in the plasma energy and the blueshift of the reflective cutoff wavelength (Figure 5), which expands the high-reflectivity region toward lower wavelengths.

In addition to the significant improvement of reflectivity, we find that the introduction of Ag increases the durability of the HfN film. In a 0.5 mol l^−1^ H_2_SO_4_ electrolyte solution, the corrosion current density of a pure HfN film is 1.81 × 10^−5^ A cm^−2^, whereas that of the doped film decreases to 1.14 × 10^−5^ A cm^−2^, only 2.4‰ of that of the pure Al film (4.69 × 10^−3^ A cm^−2^) (Supplementary Fig. S8 in Supplementary Section 11). In a 3.5 wt.% NaCl electrolyte solution, the corrosion current density of the pure HfN film is 5.83 × 10^−6^ A cm^−2^, while that of the doped film decreases to 1.90 × 10^−6^ A cm^−2^ or only 0.04‰ of that of the pure Al film (5.26 × 10^−2^ A cm^−2^) (Supplementary Fig. S9 in Supplementary Section 11). Additionally, the introduction of Ag causes an increase in the hardness of the HfN film from 22.6 to 25.4 GPa, or >50 times the hardness of an Al film (0.5 GPa) due to the solid–solution strengthening effect (Figure 4b). Additionally, during salt-bath experiments (see Supplementary Section 12), the doped film does not reveal any corrosion characteristics within 10 days (14 400 min), whereas the Al film shows significant corrosion pits in only 5 min (Supplementary Fig. S10), demonstrating that the doped film is much more corrosion resistant than Al in seawater. These results suggest that the Ag-doped HfN film not only has a high infrared reflectivity similar to Al but also has a much higher durability than Al. Therefore, it can be used as a highly effective durable infrared-reflective coating.

**Figure 6:**
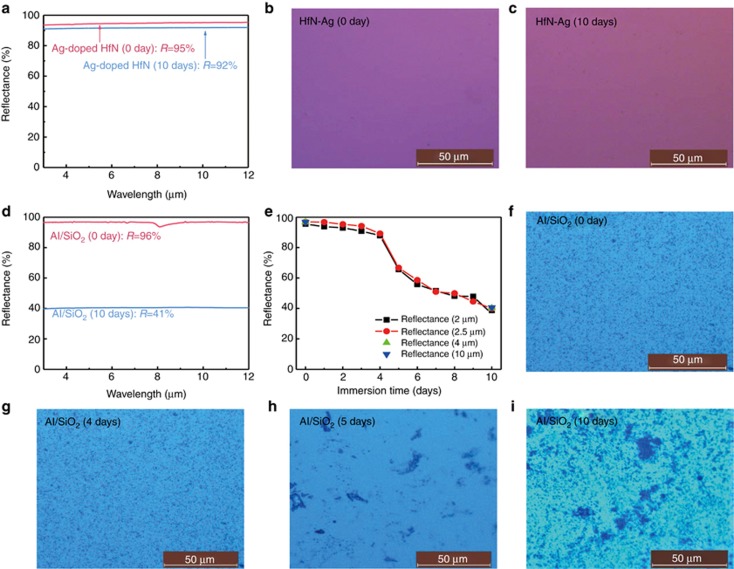
(**a**) The reflectance spectra of the Ag-doped HfN films before/after immersion in a NaCl solution at 35 °C for 10 days. (**b** and **c**) Surface of these Ag-doped HfN films before immersion (0 day) **b** and after immersion for 10 days c. (**d**) The reflectance spectra of Al/SiO_2_ film before/after immersion in the same NaCl solution for 10 days. (**e**) Reflectance of the Al/SiO_2_ films at various infrared wavelengths (2, 2.5, 4 and 10 μm) as a function of the immersion time. (**f**–**i**) Typical surface of Al/SiO_2_ films before immersion (0 day) **f**, after immersion for 4 days **g**, 5 days **h** and 10 days **i**.

The traditional method of improving the durability of an Al film is to deposit protective layers of oxides on its top. For example, SiO_2_ (230 nm) Ref. 67, TiO_2_-doped SiO_2_ (180–200 nm) Ref. 9 and SiO_2_/HfO_2_/SiO_2_ Ref. 68 films were used as the protective layers of Al films in previous studies. To compare our mirror coating with traditional ones, we deposit SiO_2_ films (approximately 200 nm) on Al films using magnetron sputtering. The infrared reflectivity and hardness of these samples (Figure 6, Table 1) are consistent with the previous experimental results^9, 67^, demonstrating the reliability of our experiments. We compare the reflectivity, stability and cost of traditional mirror coating (Al/SiO_2_ film) with our mirror coating (Ag-doped HfN film). The results are as follows. (1) The average reflectivities of an Ag-doped HfN film and Al/SiO_2_ films are 95 and 96% at 3–12 μm, respectively (Figure 6,Table 1). This fact indicates that their infrared reflectivities are very similar. (2) According to Table 1, the acid corrosion resistance, salt corrosion resistance, wear resistance and hardness of an Ag-doped HfN film are 25 times, 53 times, 10 times and 10 times those of an Al/SiO_2_ film, respectively. Additionally, the reflectivity of an Al/SiO_2_ film decreases by 55% after the 10-day salt water bath test, while the reflectivity of an Ag-doped HfN film decreases by only 3%. These results show that Ag-doped HfN films have much higher durabilities and stabilities of the reflectivity than Al/SiO_2_ films. (3) In our sputtering experiments, an Al target (diameter 60 × 3 mm) costs approximately 25 dollars, a SiO_2_ target (diameter 60 × 3 mm) costs approximately 50 dollars, a Hf target (diameter 60 × 3 mm) costs approximately 290 dollars and an Ag target (diameter 60 × 3 mm) costs approximately 150 dollars. Therefore, taking the cost of the raw materials into account, the Ag-doped HfN film is more expensive than the Al/SiO_2_ film. However, the Ag-doped HfN film only needs to deposit a one-layer film, which is easier than the two-step preparation of the two-layer Al/SiO_2_ film. Moreover, the Ag-doped HfN films have much higher durabilities and stabilities. Thus we believe that the cost of the two mirror coatings is very similar. These results suggest that the Al/SiO_2_ films have slightly better reflectivities and a lower cost than the Ag-doped HfN films, but their durabilities and stabilities of the reflectivity are far lower than those of Ag-doped HfN films. Thus the traditional mirror coating is more suitable for moderate environments, while our mirror coating obtained by the new strategy is more suitable for harsh environments, such as those with dust, windblown sand, moisture, acid rain and salt fog.

## Conclusions

We propose an alternative design approach of ‘first durability and then reflectivity’ to achieve the goal of both high durability and reflectivity. We recommend transition metal compounds, *TMR* (*TM*=Ti, Zr, Hf, V, Nb, Ta, Cr, Mo, W; *R*=B, C, N), as the durable base material and then improve its reflectivity to achieve the integration of high durability and reflectivity. The two validation experiments on HfN prove that our new strategy and approaches work as expected. The coatings thus obtained not only have an infrared reflectivity performance close to that of aluminum but also have a far better durability. The traditional mirror coating (e.g., Al/SiO_2_ films) is more suitable for moderate environments, while our mirror coating that is obtained by the new strategy (e.g., Ag-doped HfN film) is more suitable for harsh environments, such as those with dust, windblown sand, moisture, acid rain and salt fog. This research opens up whole new areas in developing durable infrared-reflective coatings. HfN is only 1 of the 27 *TMR* transition metal compounds; thus this study can be considered one point in the whole spectrum of possibilities, that is, zero dimension. By substituting any other transition metal for Hf and/or replacing N with B and C, a two-dimensional study can be mapped out. Furthermore, the inherent properties of each *TMR* can be improved by modulating its structure (multilayering, alloying, nanocompositing, etc.); thus new three-dimensional studies may emerge in this area. As such, new materials, new functions and new mechanisms are expected to be developed. This study has ushered the transition metal compounds from hard protective coatings (e.g., cutting-tool coatings) into a completely new area of optical coatings. This rejuvenates the study of the transition metal compounds.

## Figures and Tables

**Table 1:** Stability of reflectivity, hardness, wear rate and corrosion current for Ag-doped HfN and Al/SiO_2_ films

	Ag-doped HfN	Al/SiO_2_	Comparison
Reflectance (3–12 μm)			
As-deposited	95%	96%	1% lower than Al/SiO_2_
After immersion in a NaCl solution at 35 °C for 10 days	92%	41%	51% higher than Al/SiO_2_
			
Hardness (GPa)	25.4	2.5	10:1 (10 times)
Wear rate (10^−5^ mm^3^ N^−1^m^−1^)	1.03	11.76	1:10 (10 times)

Corrosion current (A cm^−2^)			
0.5 mol l^−1^ H_2_SO_4_	1.14E-05	2.90E-04	1:25 (25 times)
3.5 wt.% NaCl	1.90E-06	1.02E-04	1:53 (53 times)
