# Supplementary material for: New design for highly durable infrared-reflective coatings
Source: Light Sci Appl. 2018 Apr 6;7:17175–. doi: 10.1038/lsa.2017.175 (PMC6060051; doi:10.1038/lsa.2017.175)
Supplement: Supplementary Information [file lsa2017175x1.pdf]

SUPPLEMENTARY INFORMATION

New design for highly durable infrared-reflective coatings

Chaoquan Hu[1]*, Jian Liu[1], Jianbo Wang[2], Zhiqing Gu[1], Chao Li[1], Qian Li[1], Yuankai Li[1], Sam Zhang[3]*, Chaobin Bi[1], Xiaofeng Fan[1]* and Weitao Zheng[1,4]*

[1] *State Key Laboratory of Superhard Materials, Key Laboratory of Automobile Materials of MOE, and School of Materials Science and Engineering, Jilin University, Changchun 130012, China*
[2] *School of Science, Changchun University of Science and Technology, Changchun 130022, China*
[3] *Faculty of Materials and Energy, Southwest University, Chongqing 400715, China*
[4] *State Key Laboratory of Automotive Simulation and Control, Jilin University, Changchun 130025, China*

***Corresponding authors.** E-mail: cqhu@jlu.edu.cn (C. Q. Hu); msyzhang@live.com (S. Zhang); xffan@jlu.edu.cn (X. F. Fan); wtzheng@jlu.edu.cn (W. T. Zheng);

This file includes:

Figures S1-S11

Tables S1-S2

References 1-6

**Sections**	**Page**

Section 1 Two-stage electron localization of HfN_x films with stoichiometry 1

Section 2 Two-stage structural evolution of HfN_x films with stoichiometry 3

Section 3 Electronic properties of stoichiometric HfN 4

Section 4 Contribution of Hf vacancies and phase transformation to electron localization 6

Section 5 Effect of number of layers, optical thickness and refractive index on reflectivity enhancement 11

Section 6 Refractive index and thickness of the HfN_x multilayer films 12

Section 7 Preparation conditions for the HfN_x multilayer films 13

Section 8 Potentiodynamic polarization curves for the HfN_x multilayer and Al films 14

Section 9 Salt bath experiments for the HfN_x multilayer and Al films 16

Section 10 Electronic properties of HfN-Ag 17

Section 11 Potentiodynamic polarization curves for HfN, HfN-Ag and Al films 19

Section 12 Salt bath experiments for HfN-Ag and Al films 21

Section 13 Salt bath experiments for Al/SiO_2 films 22

References 23

Section 1 Two-stage electron localization of HfN_x films with stoichiometry

Fig. 2a shows the electron concentrations of HfN_x films with different stoichiometry x, in which the electron concentration drops sharply as x increases. The electron concentration is 1.46×10^{22} cm^{-3} when x is about 1:1 (a measuring value of 1.039), which is very close to that of Au (same order of magnitude in value: n_{Au} = 5.90×10^{22} cm^{-3}) (Ref. 1), indicating the near-stoichiometric HfN_x films is metallic in nature. However, when x increases to 4:3 (a measuring value of 1.334), the electron concentration decreases by 13 orders of magnitude to 1.67×10^{9} cm^{-3}, which is very close to the intrinsic carrier concentration of Si at room temperature (n_{Si} = 1.45×10^{10} cm^{-3}) (Ref. 2), meaning that the near Hf_3N_4 films exhibit apparent semiconductor characteristics. Fig. 2b shows the electrical resistivity of HfN_x films with different x, wherein the film with x = 1.039 has a low electrical resistivity of 110 μΩ cm, consistent with electrical resistivity of metals, typically on the order of 1-10^{3} μΩ cm (Ref. 3), confirming that the nearly stoichiometric film is basically metallic. However, when x increases to 1.334, electrical resistivity of the film becomes as high as 4.50×10^{4} μΩ cm, increased by nearly 3 orders of magnitude and behaves semiconductor-like. To confirm the semiconductor characteristic, the Tauc plotting is employed to determine the optical gap of the $HfN_{1.334}$ film with a result of about 2.50 eV (Fig. 2c). The increase of electrical resistivity, formation of band gap and decrease of electron concentration all congregate to point to the same conclusion: as x increases from 1/1 to 4/3 HfN_x films change from metal to semiconductor where free electrons become localized.

It is worth noting that the whole electron localization process is not “uniform”,

but rather “slow” at first and accelerated with further increase of x. In Figs. 2a and b, as x increases from 1.039 to 1.165, electron concentration decreases slowly from 1.46×10^{22} to 6.01×10^{20} cm^{-3}. Meanwhile, the electrical resistivity gradually increases from 110 to 636 μΩ cm. After 1.195, however, as x further increases to 1.334, electron concentration decreases sharply from 5.59×10^{20} to 1.67×10^{10} cm^{-3}, a drop of 10 orders of magnitude. The electrical resistivity sharply increases 30 times from 1.51×10^{3} to 4.50×10^{4} μΩ cm. These results demonstrate that the electron localization in HfN_x films experiences two stages as x increases: x = 1.039-1.165, electrons are “gradually” localized; x = 1.195-1.334, a small increase in x causes large number of electrons localized, resulting in the films losing their metallic characteristic and “rapidly” transform into semiconductors. The large difference between the transition speeds implies that there are different mechanisms behind the two-stage electron localization. This is illustrated in the next section.

Section 2 Two-stage structural evolution of HfN_x films with stoichiometry

To better understand the mechanism of the two-stage electron localization, the influence of stoichiometry x on the structure of the HfN_x films is studied via HRTEM, SAED, Raman, XRD and XPS[4]. All the results are in support of each other, proving that the structures are different in the two stages. In the region of x = 1.039-1.165, increasing of x is compensated by formation of more and more Hf vacancies while the rocksalt structure remains. In the region of x = 1.195-1.334, further increase in x can't be balance out by Hf vacancies, formation of c-Hf_3N_4 phase takes place. At N/Hf = 4:3 or x reaches 1.334, this phase transition completes.

Section 3 Electronic properties of stoichiometric HfN

The pristine HfN has the NaCl-type structure with space group Fm-3m (Fig. S1a). The calculated lattice constant is 4.53 Å and is similar to the experimental value of 4.58 Å. From the band structure, this is metallic of good conductivity (Fig. S1b). The type of carriers is the hole-like. From the density of states (DOS, Fig. S1d), the energy region between -9 eV and -3 eV is characterized by the hybridization of the d-orbitals of Hf and p-orbitals of N. The region from -3 eV to Fermi level is controlled mainly by the d-electrons of Hf. By analyzing the DOS and band structure of HfN, the band states from -3 eV to Fermi level is with high dispersion. From the distribution of electron charge density (Fig. S1c), the free electrons are localized due to the contribution of excess Hf_d electrons partially in the tetrahedral interstitial sites formed by four near-neighbor nitrogen ions.

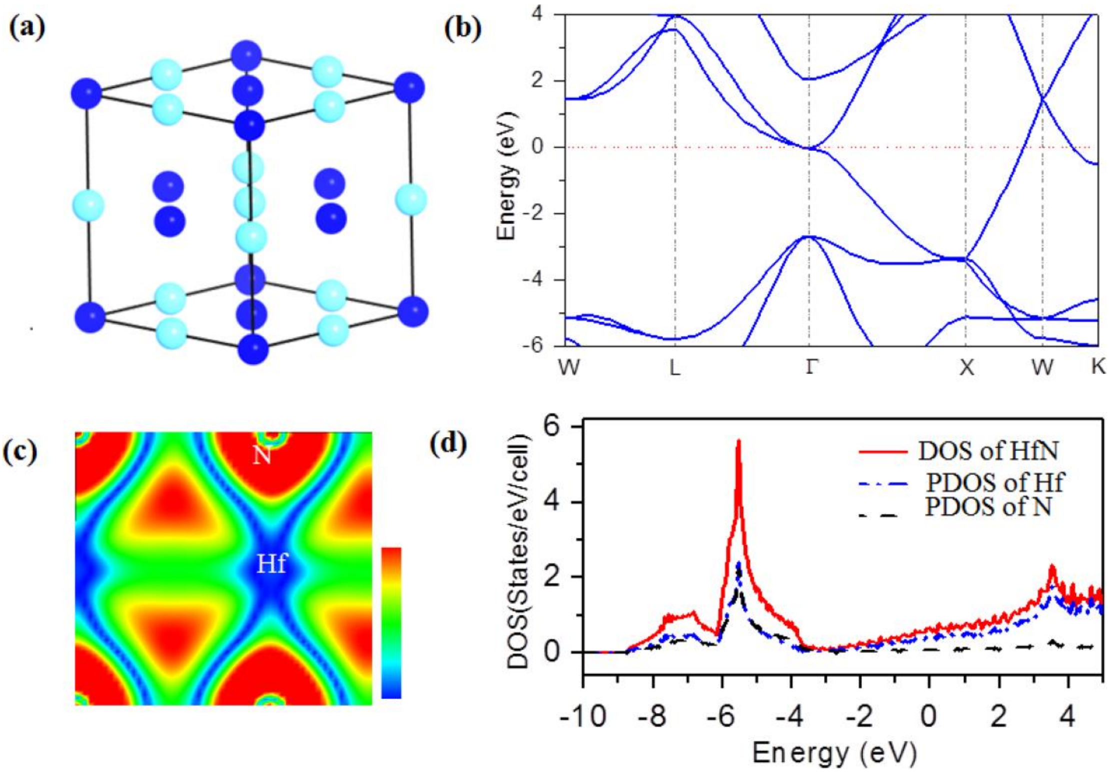

Figure S1. Schematic representations of structure (a), band structure (b), distribution of electron charge density difference in (1 1 0) plane (c), and density of states (DOS) and partial density of states (PDOS) (d) of stoichiometric HfN with NaCl-type structure

Section 4 Contribution of Hf vacancies and phase transformation to electron localization

In over-stoichiometric HfN_x film, the main point defects are hafnium vacancies (V_{Hf}). The structure of $HfN_{1.143}$ is built by a 2 × 2 × 2 primitive cell of HfN with one V_{Hf} (Fig. S2a). The point defect V_{Hf} doesn't introduce the localized magnetic moment and result in the spin-polarization. From the density of states (DOS in Fig. S2b), as the Hf vacancy is introduced, the peak around -5.20 eV has an obvious up-shift with formation of some new added states at around -2.50 eV. From the partial DOS (PDOS), these newly added states arise from the contribution of N near V_{Hf}. Fig. S3c shows the N_1*s*-Hf_4*f* binding-energy difference obtained experimentally by XPS core-level spectra for the HfN_x films with different *x*. Obviously, with the increase of *x* in Stage I, the difference gradually decreases, indicating that the formation of Hf vacancies causes free electrons continuously transferred from Hf to N atoms[5]. A good agreement between the measured XPS and calculated DOS proves that the formation of Hf vacancies enables the localization of part of free electrons around Fermi level and promotes the new localized states from N_p in this stage.

With increase *x*, more Hf vacancies form and then merge, resulting in transforming of the structure of HfN_x from NaCl-type (δ-HfN) to Th_3P_4-type (c-Hf_3N_4). The c-Hf_3N_4 with space group I*43d* is considered by a 28-atom supercell (Fig. S2c). The calculated lattice constant of c-Hf_3N_4 is 6.69 Å, very close to that of the experiments (~6.67 Å). In DOS of c-Hf_3N_4, the valance band from -7 eV to 0 eV is mainly from the hybridization of the Hf_d with N_p orbitals. In comparison to δ-HfN, the DOS near the Fermi level of c-Hf_3N_4 disappeared, accompanied by the

presence of a band gap. Full depletion of free-d electrons and band gap opening clearly show the phase transformation from δ-HfN to c-Hf_3N_4 inducing the formation of semiconductor. The complete localization of Hf_d electrons induces a transition of the coordination number of Hf from 6 to 8 atoms. This results in the formation of the typical chemical bonds with semi-ionic characteristic between Hf and N atoms, which is confirmed by the electron charge density difference (Fig. S2d).

At near-stoichiometric (x = 1.039) (Fig. S3f), there are two peaks in the valence-band spectrum, a relatively weak peak located near the Fermi level (0 ~ -2 eV) and another dominant centered at -6.00 eV. Compared with the calculated DOS (Fig. S3d), these two peaks arise from the states of Hf_d and the hybridized states of N_p and Hf_d, respectively. In c-Hf_3N_4 film (x = 1.334), the dominant peak appears near -5.50 eV, shifting about 0.50 eV towards Fermi level. At the same time, the weak peak near the Fermi level disappeared and a band gap of about 2.00 eV emerges. The difference in valence-band spectra between δ-$HfN_{1.039}$ and c-$HfN_{1.334}$ is obtained and shown in Fig. S3f. It can be clearly seen that the original electrons of 0 eV ~ -2 eV near E_F in δ-$HfN_{1.039}$ are transferred to the newly hybridized states around -2.00 eV ~ -5.50 eV in c-$HfN_{1.334}$. Furthermore, XPS core-level results (Fig. S3c) show that as the δ-HfN phase is gradually transformed into the c-Hf_3N_4 phase with increasing x from 1.195 to 1.334, N 1s-Hf 4f binding energy difference rapidly decreases, indicating the transfer of free electrons accelerates from Hf to N atoms[5]. This is in good agreement with our calculations and expectation: the phase transition causes free electrons near Fermi level localized fully, new hybridized states from Hf_d and N_p

are created in Stage II ($1.195 \le x \le 1.334$).

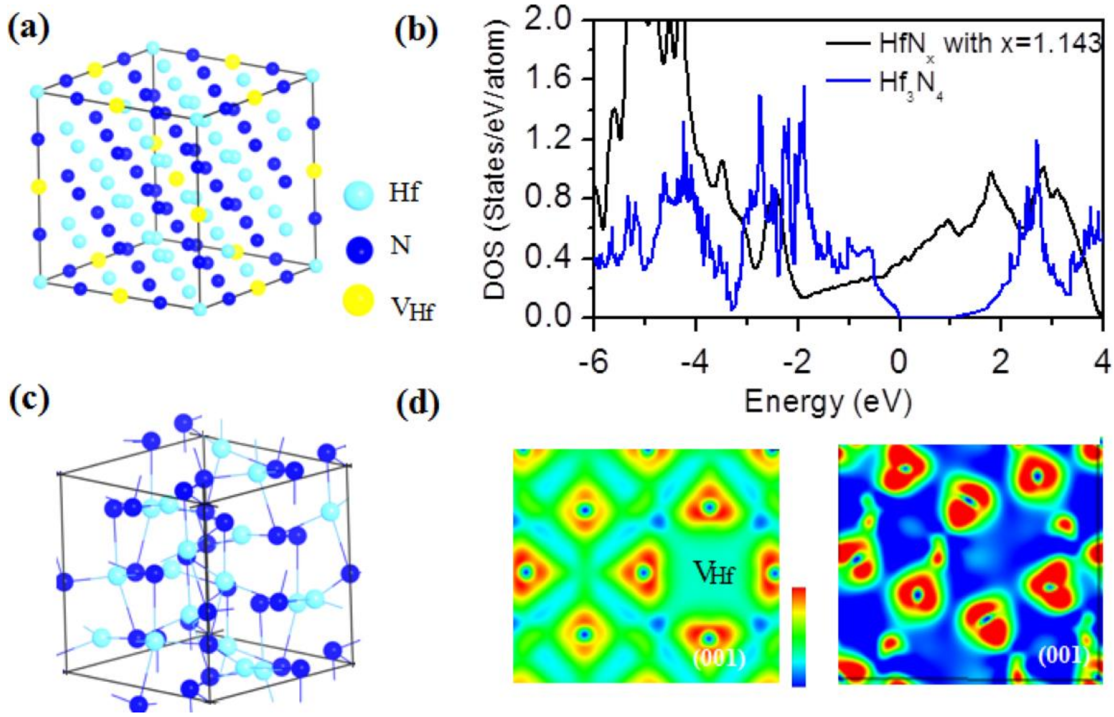

Figure S2. Schematic representations of the structure of V_{Hf}-containing NaCl-type $HfN_{1.143}$ (a) and Th_3P_4-type Hf_3N_4 (c), density of states of $HfN_{1.143}$ and Hf_3N_4 (b), and distribution of electron charge density difference in (100) plane of V_{Hf}-containing $HfN_{1.066}$ and Hf_3N_4 (d)

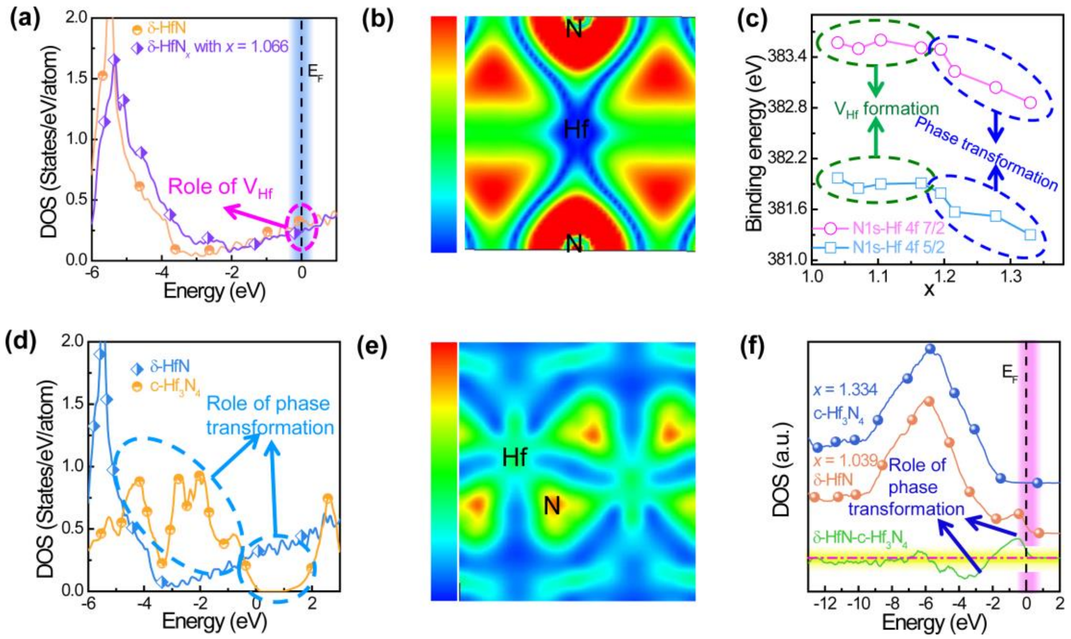

Figure S3. Density of states (DOS) of NaCl-type stoichiometric HfN and HfN_x with x = 1.066 (a), distribution of electron density differences of NaCl-type HfN (b) and measured XPS core-level spectra of HfN_x films with different x (c), in which the contribution of Hf vacancies to electron localization is revealed. DOS of NaCl-type HfN and Th_3P_4-type c-Hf_3N_4 (d), distribution of electron density differences of Th_3P_4-type c-Hf_3N_4 in (100) plane (e) and measured XPS valence-band of HfN_x films with different x (f), where the contribution of phase transition to electron localization is shown.

Section 5 Effect of number of layers, optical thickness and refractive index on reflectivity enhancement

To increase the reflectivity, a certain number of repeating a low-refractive-index transparent HfN_x layer (*LT*) and a high-refractive-index transparent HfN_x layer (*HT*) is deposited on a base of opaque metallic HfN_x layer (*OM*) to form a multilayer $OM/(LT/HT)_z$ where z is the total number of repeating layers. The resultant reflectivity depends on each layer's refractive index (n), thickness (d) and total number of layers (z). According to the principle of optical interference[6], to achieve high reflectivity at a targeted wavelength (λ_0), the optical thickness or the product of n and d of layer 1 and layer 2 should equal to a quarter of the target wavelength, that is, $n_1d_1 = n_2d_2 = \lambda_0/4$. Meanwhile, the greater the difference between n_1 and n_2, the more pronounced the enhancement. And, the more the repeating layers (i.e., the value of z), the better the enhancement. However, increasing z increases the deposition time and processing difficulty. After certain z, the benefit brings by z tapers off. Everything considered, we set z = 6, i.e., *(LT/HT)* is repeated for 6 times on the base of *OM*, with vastly different refractive indexes. The thickness and refractive index of each layer are illustrated in the next section.

Section 6 Refractive index and thickness of the HfN_x multilayer films

To prove the multilayer design can achieve high reflectivity at any wavelength, we choose, as the target wavelengths λ_0, 1900 nm in the near-infrared and 4100 nm in mid-infrared bands to carry out the validation tests (on *OM* layer with 1000 nm in thickness at $x = 1.039$). For the multilayers with $\lambda_0 = 1900$ nm, the refractive indices of *LT* and *HT* layers are $n_1 = 2.08$ at $x = 1.396$ and $n_2 = 2.69$ at $x = 1.342$, respectively. According to $n_1 d_1 = n_2 d_2 = \lambda_0/4$, when $\lambda_0 = 1900$ nm, the thickness of the two layers are $d_1 = 228$ nm and $d_2 = 177$ nm. Similarly, for $\lambda_0 = 4100$ nm, $n_1 = 2.17$ at $x = 1.383$ and $n_2 = 2.78$ at $x = 1.334$, d_1 and d_2 are 472 nm and 369 nm, respectively.

Section 7 Preparation conditions for the HfN_x multilayer films

During the magnetron sputtering deposition, we obtained HfN_x thin films in three optical characteristics by controlling the flow rate of nitrogen and argon: an opaque metal (*OM*, x measured as 1.039), a high-refractive-index transparent semiconductor (*HT*, x measured as 1.334-1.342, n = 2.78-2.69), a low-refractive-index transparent semiconductor (*LT*, x measured as 1.383-1.396, n = 2.17-2.08). We stacked these three films and prepared two kinds of HfN_x multilayer films with λ_0 = 1900 and 4100 nm. The nitrogen flow rate (F_{N2}), argon flow rate (F_{Ar}) and deposition time (t) in the preparation process are listed in the following tables.

Table S1: Preparation conditions of the multilayer film with λ_0 = 1900 nm.

	F_{N2}/sccm	F_{Ar}/sccm	t/min
OM	3.6	80.0	90
LT	40	0	300
HT	42.4	20.0	64
Multilayer structure: OM/(LT/HT)$_z$, z = 6			

Table S2: Preparation conditions of the multilayer film with λ_0 = 4100 nm.

	F_{N2}/sccm	F_{Ar}/sccm	t/min
OM	3.6	80.0	90
LT	38.2	3.0	300
HT	28.3	20.0	100
Multilayer structure: OM/(LT/HT)$_z$, z = 6			

Section 8 Potentiodynamic polarization curves for the HfN_x multilayer and Al films

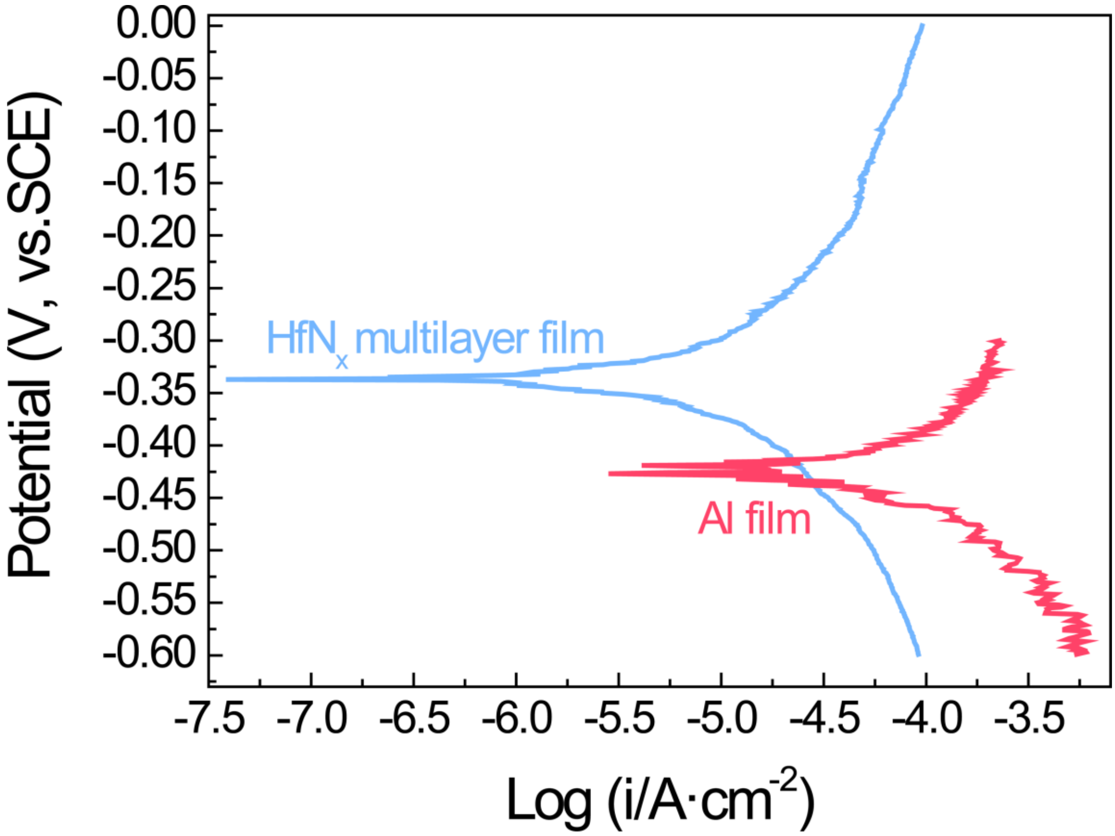

Figure S4. Potentiodynamic polarization curves for the HfN_x multilayer and Al films in a 0.5 mol/L H_2SO_4 solution.

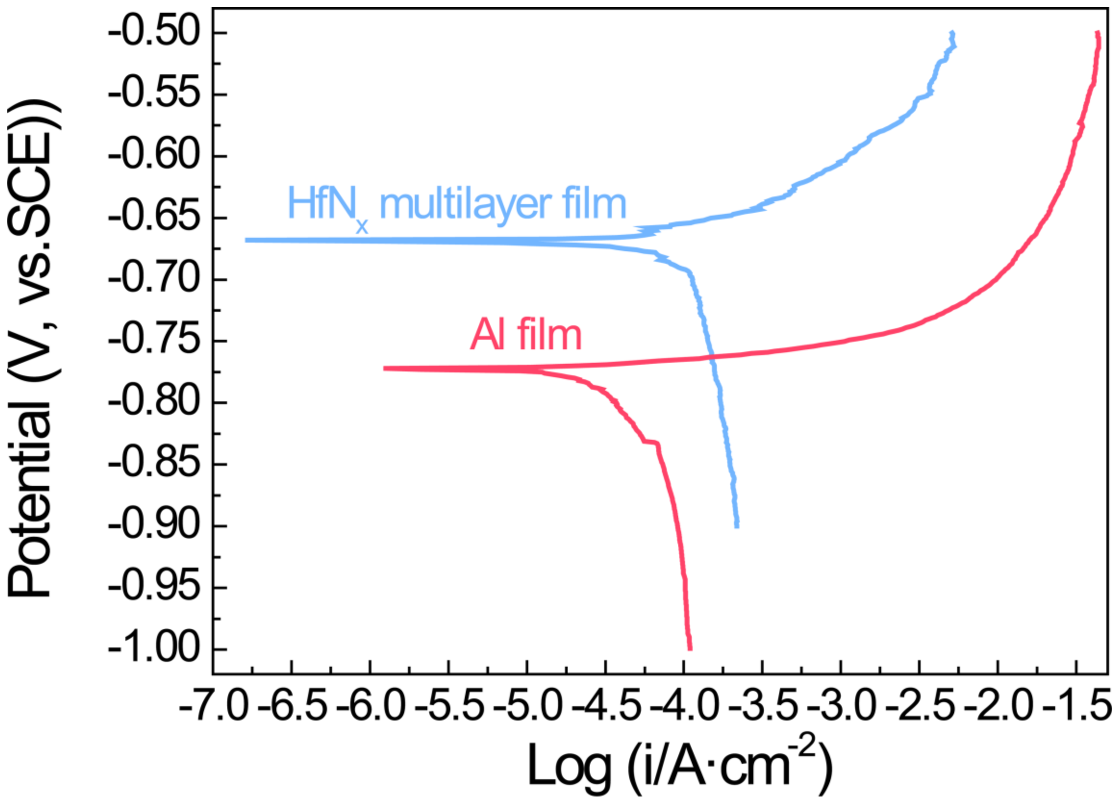

Figure S5. Potentiodynamic polarization curves for the HfN_x multilayer and Al films in a 3.5 wt.% NaCl solution with deionized water.

Section 9 Salt bath experiments for the HfN_x multilayer and Al films

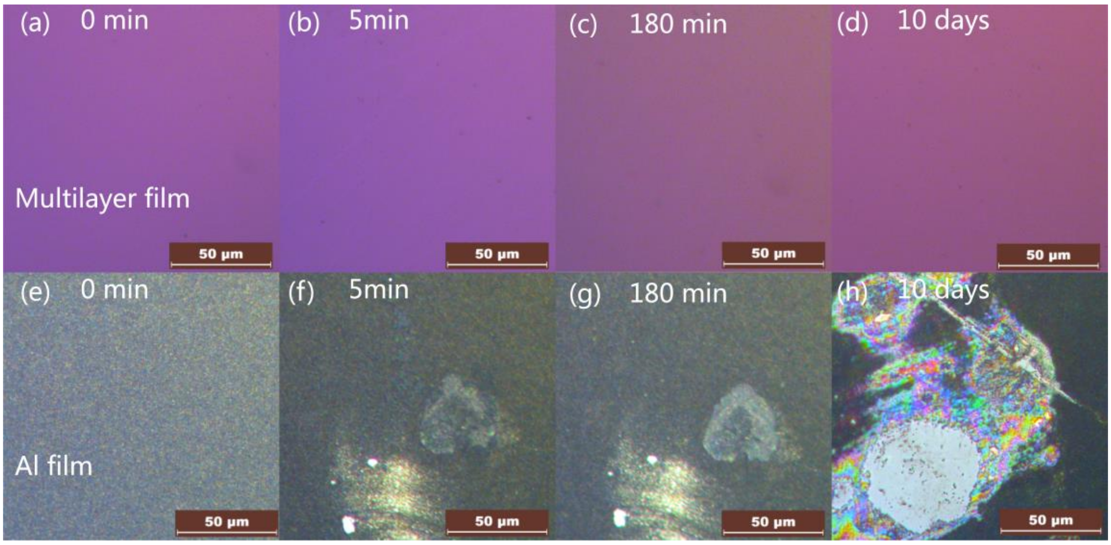

Figure S6. Corroded surface of the HfN_x multilayer and Al films after immersion in a NaCl solution at 35 °C for different times: 0 min (a, e), 5 min (b, f), 180 min (c, g) and 10 days (14,400 min) (d, h).

Section 10 Electronic properties of HfN-Ag

A structural model of 8 atoms in which one Hf atom is replaced by one Ag atom is built to reveal the effect of Ag doping on the electronic properties of HfN (Fig. S7a). From the band structure (Fig. S7b), the introduction of Ag into the lattice of HfN doesn't induce any localized bands/states near Fermi level. The density of states near Fermi level doesn’t increase obviously. This means the Ag doping have a weak effect on the electron conductivity. From the PDOS (Fig. S7d), the Ag doping induces new states from -3.50 eV to -2 eV. The quasi-free electrons from these new states are expected to have important contributions to optical reflectivity. From the electron charge density difference (Fig. S7c), the electrons from Ag are transfer to the nearby N ions with the polarization of N ions.

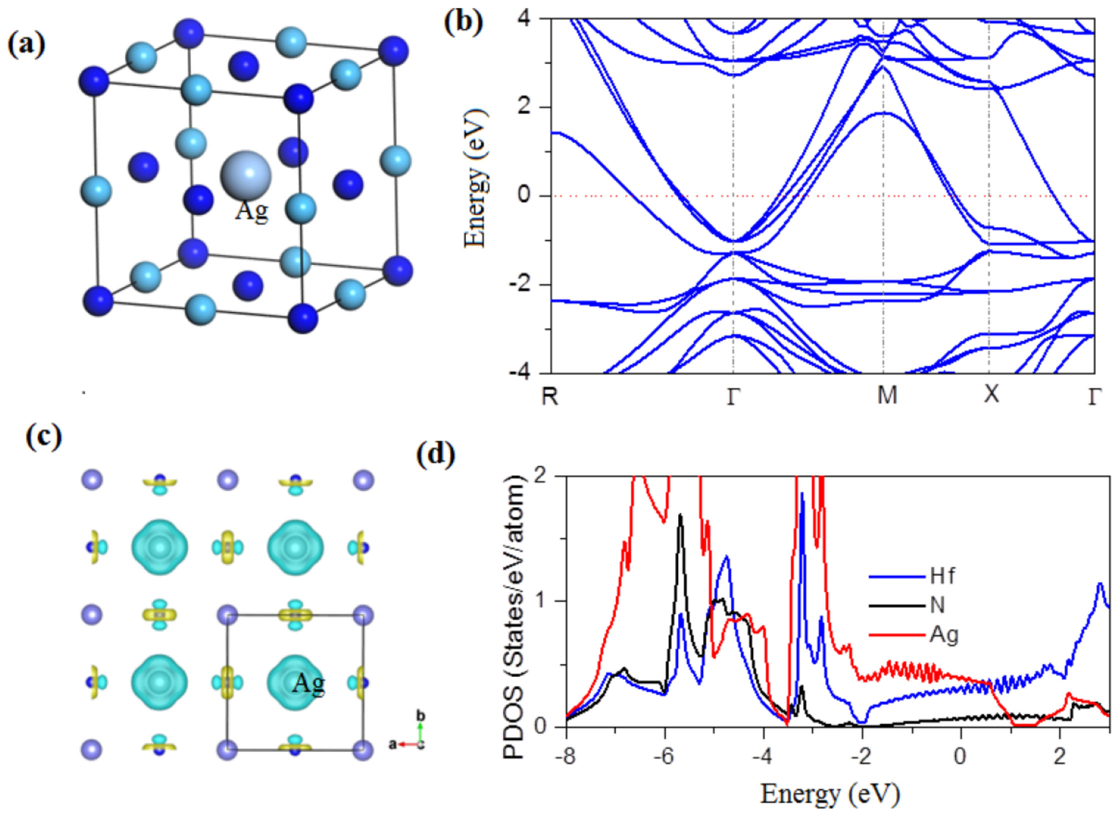

Figure S7. Schematic representations of structure (a), band structure (b), distribution of electron charge density difference in (1 1 0) plane (c), and partial density of states (PDOS) (d) of Ag-doped HfN with NaCl-type structure. Note that the yellow and blue correspond to the charge accumulation and charge depletion, respectively.

Section 11 Potentiodynamic polarization curves for HfN, HfN-Ag and Al films

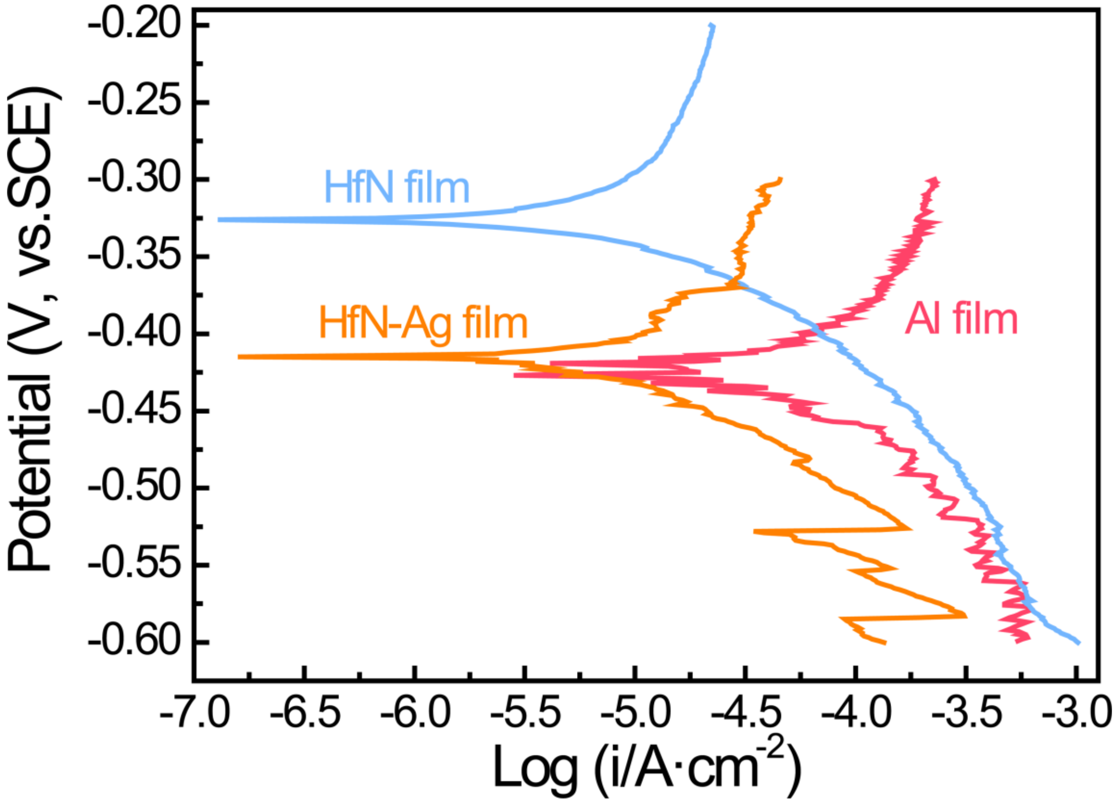

Figure S8. Potentiodynamic polarization curves for HfN, HfN-Ag and Al films in a 0.5 mol/L H_2SO_4 solution.

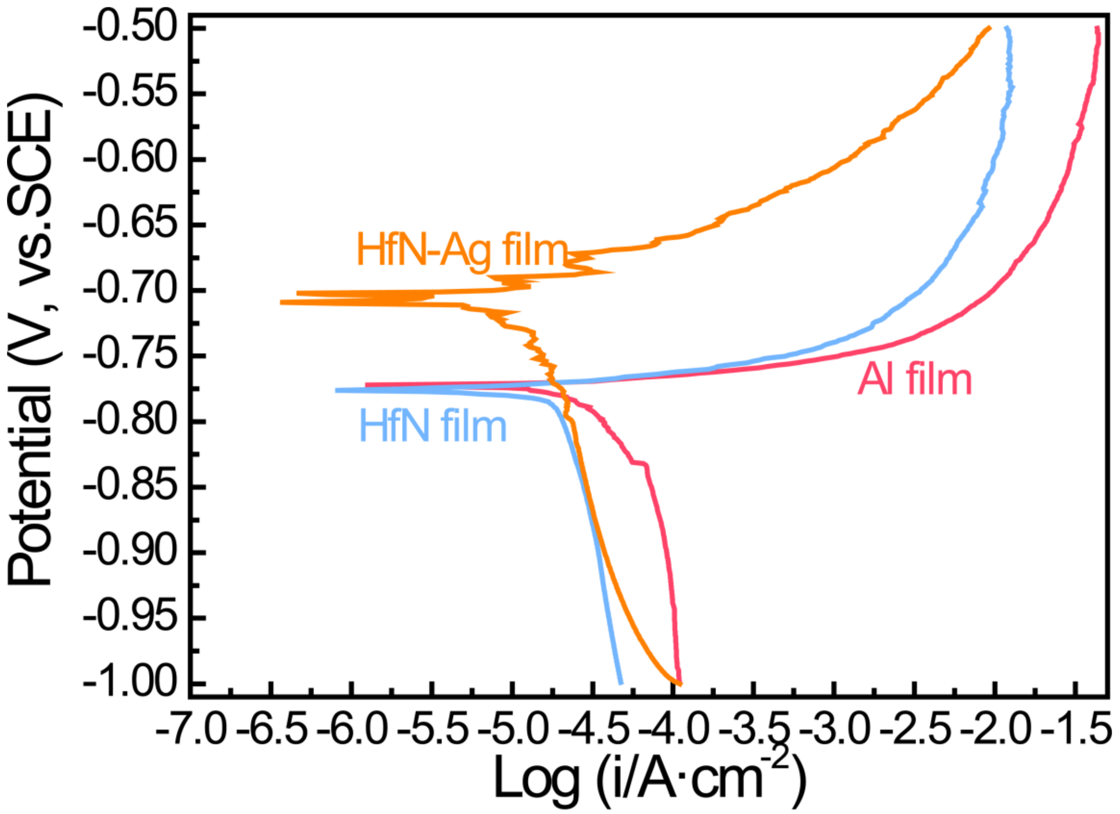

Figure S9. Potentiodynamic polarization curves for HfN, HfN-Ag and Al films in a 3.5 wt.% NaCl solution with deionized water.

Section 12 Salt bath experiments for HfN-Ag and Al films

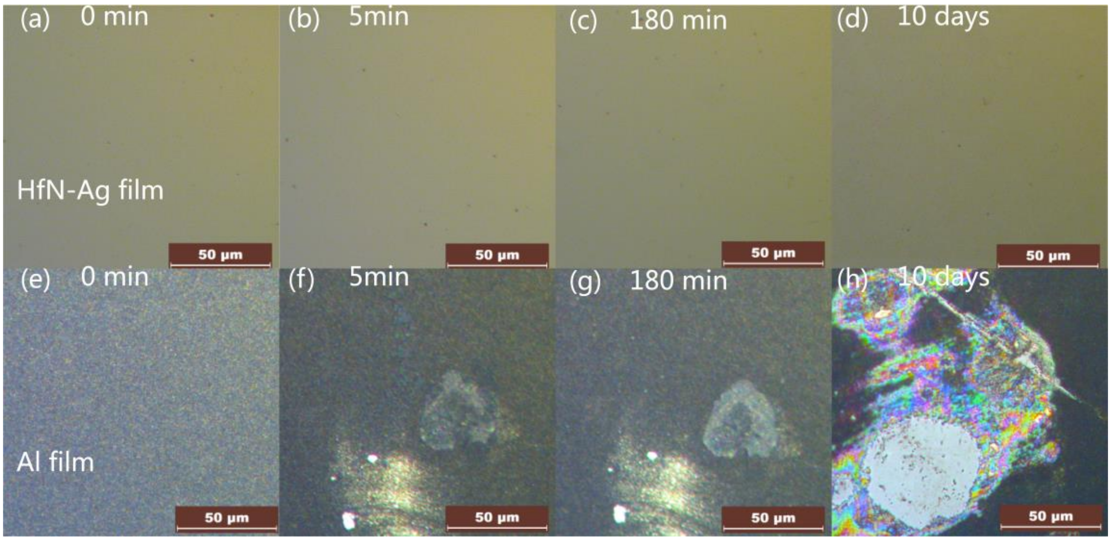

Figure S10. Corroded surface of the HfN-Ag and Al films after immersion in a NaCl solution at 35 °C for different times: 0 min (a, e), 5 min (b, f), 180 min (c, g) and 10 days (14,400 min) (d, h).

Section 13 Salt bath experiments for Al/SiO_2 films

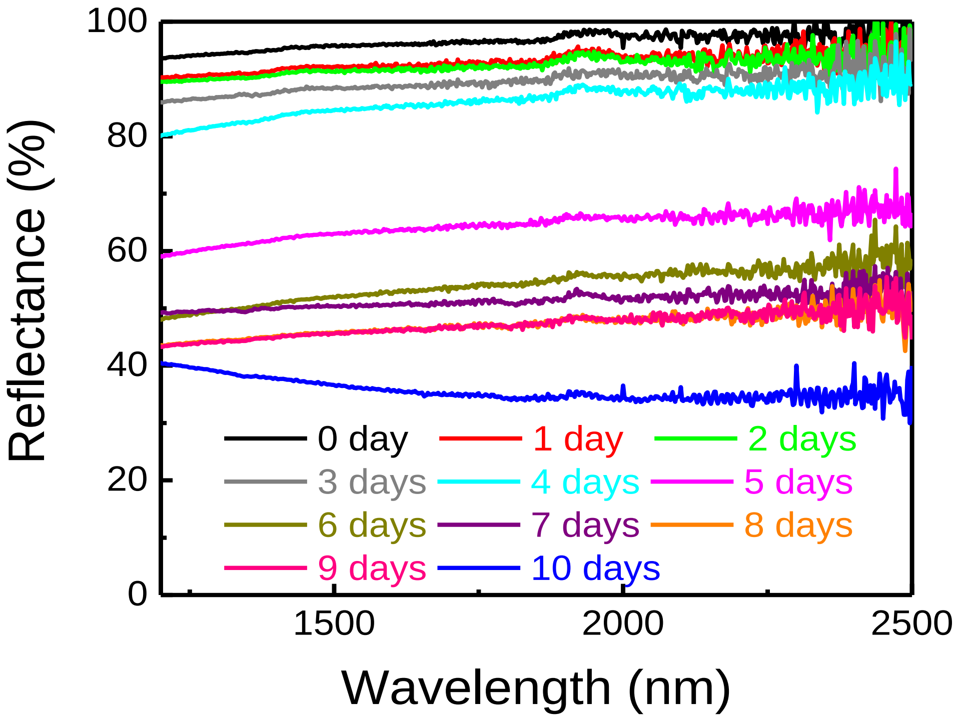

Figure S11. The reflectance spectra of Al/SiO_2 films after immersion in a NaCl solution at 35 °C for different time: 0 day (before immersion) - 10 days.

References

1. Ashcroft NW, Mermin ND. *Solid State Physics.* New York: Holt, Rinehart and Winston; 1976.
2. Sproul AB, Green MA. Improved value for the silicon intrinsic carrier concentration from 275 to 375 K. *J Appl Phys* 1991; **70**: 846-854.
3. Callister WD. *Fundamentals of Materials Science Engineering, Fifth Edition: An Interactive*. Beijing: Chemical Engineering Press; 2004.
4. Gu ZQ, Hu CQ, Huang HH, Zhang S, Fan XF *et al.* Identification and thermodynamic mechanism of the phase transition in hafnium nitride films. *Acta Mater* 2015; **90**: 59-68.
5. Prieto P, Galan L, Sanz JM. Electronic structure of insulating zirconium nitride. *Phys Rev B* 1993; **47**: 1613.
6. Pedrotti LS. Basic physical optics. In: Roychoudhuri C (ed.) *Fundamentals of Photonics.* Bellingham: SPIE Press; 2008**:** 127-138.
